# A prospective study of a single-incision sling at the time of robotic sacrocolpopexy

**DOI:** 10.1007/s00192-014-2432-8

**Published:** 2014-06-04

**Authors:** Carolyn Botros, Christa Lewis, Patrick Culligan, Charbel Salamon

**Affiliations:** 1 Division of Urogynecology and Reconstructive Pelvic Surgery, Atlantic Health System, Morristown, NJ USA; 2 435 South Street, Suite 370, Morristown, NJ 07960 USA

**Keywords:** Pelvic organ prolapse, Robotic sacrocolpopexy, Single-incision sling, Stress urinary incontinence

## Abstract

**Introduction and hypothesis:**

The objective of this study was to evaluate the efficacy and safety of the Miniarc Precise® single-incision sling (American Medical Systems, Minnetonka, MN, USA) placed at the time of a robotic sacrocolpopexy.

**Methods:**

This was a prospective study of a single-incision suburethral sling placed at the time of robotic sacrocolpopexy in women with stress urinary incontinence (SUI) and pelvic organ prolapse. Primary outcome measure was cure at 1 year, defined objectively by a negative cough stress test (CST) and subjectively by a score of “0 or 1” on question 17 of the Pelvic Floor Distress Inventory (PFDI-20): “Do you experience urine leakage related to coughing/sneezing/laughing?” Secondary outcome measures included the change in Urinary Distress Inventory (UDI-6) and Urinary Impact Questionnaire (UIQ-7) scores at 1 year. All sling-related complications were reported. Paired Student’s *t* test and the Wilcoxon signed-rank test were used for statistical analysis.

**Results:**

One hundred and one patients were included between August 2010 and July 2012. One-year follow-up was available for 97 out of 101 patients (96 %). Objective cure was 90 % and subjective cure was 87 %. Baseline UDI-6 scores improved from 34.8 ± 25.1 to 6.7 ± 11.2 at 1 year (*p* < 0.001). Similarly, UIQ-7 scores improved from 21.1 ± 22.8 to 2.4 ± 8.2 at 1 year (*p* < 0.001). There were no intraoperative cystotomies, no mesh erosions, no sling revisions, and no cases of urinary retention. The retreatment rate for persistent SUI was 8 % (8 out of 97).

**Conclusions:**

The addition of a single-incision suburethral sling at the time of robotic sacrocolpopexy in women with SUI resulted in an 87 % cure rate at 1 year.

## Introduction

Stress urinary incontinence (SUI) is a common condition that affects up to 35 % of women above the age of 50 [1]. The majority of women presenting with pelvic organ prolapse have coexisting SUI [2]. Furthermore, prolapse repair is associated with new onset stress incontinence in a large proportion (up to 40 %) of previously continent patients [3, 4]. The Colpopexy and Urinary Reduction Efforts (CARE) trial concluded that the routine addition of Burch colposuspension to abdominal sacrocolpopexy significantly decreased the incidence of de novo SUI [3]. Similar results were shown with the placement of a suburethral sling at the time of prolapse repair in the Outcomes Following Vaginal Prolapse and Midurethral Sling (OPUS) trial [4].

Retropubic suburethral slings are associated with some risk of retropubic hematoma, bladder and bowel perforation, urinary retention, and long-term voiding dysfunction; including urgency, frequency, and incomplete bladder emptying [5, 6]. The risk of developing these symptoms may be higher in patients with preoperative obstructive voiding. Alternatively, transobturator slings offered a lower rate of the above-mentioned complications, but have added the risks of inner thigh pain, vaginal sulci pain, and mesh exposure [7–9]. Single-incision slings were developed to further reduce sling-related complications; unfortunately, their efficacy has been questioned owing to less than desirable results in early trials [10]. However, single-incision slings have different designs and anchoring mechanisms and would be expected to have different success rates. The FDA’s 2013 publication: “Considerations about surgical mesh for SUI” stated that “Additional studies may help the agency to better understand the safety and effectiveness of these (mini-slings) devices” [11].

A well-conducted, single-arm, multicenter prospective study evaluated the Miniarc Precise® single-incision sling (American Medical Systems, Minnetonka, MN, USA) and found it to be highly efficacious (80 % objective cure) with a low complication rate [12].

Prior studies focused on the efficacy of the single-incision sling as a standalone therapy for isolated SUI. The literature is sparse regarding single-incision sling placement at the time of prolapse repair and there are no studies specifically evaluating the Miniarc Precise® single-incision sling at the time of robotic sacrocolpopexy.

At our center, one of the attending surgeons (C.S.) started offering the Miniarc Precise® single-incision suburethral sling to the majority of patients with pelvic organ prolapse undergoing a robotic sacrocolpopexy. The objective of this study was to prospectively assess the 1-year outcomes of the Miniarc Precise® single-incision suburethral sling placed at the time of robotic sacrocolpopexy.

## Materials and methods

This was a prospective study, approved by the Atlantic Health Institutional Review Board (R12-09-001) and listed on www.clinicaltrials.gov (NCT01982188). The study included all consecutive patients who underwent placement of a single-incision suburethral sling (Miniarc Precise®, by American Medical Systems, Minnetonka, MN, USA) at the time of robotic sacrocolpopexy. Inclusion criteria were stage II to IV pelvic organ prolapse and a positive reduction cough stress test (CST) in women with or without a complaint of urinary incontinence. Patients with previous surgery for SUI were excluded from the study. At the conclusion of the robotic sacrocolpopexy, the sling was placed through a 2-cm vertical suburethral vaginal incision. Midurethral tunnels were created bilaterally following the same trajectory as a transobturator sling dissection and the sling was inserted aiming toward the notch under the ipsilateral adductor longus tendon. The anchoring tip rested in the internal obturator muscle at its insertion site into the pubic ramus without traversing the obturator membrane or any of the inner thigh muscles. The sling mesh laid flat against the mid-urethra without tension or any intervening space.

Outcome measures were evaluated at baseline and 1 year postoperatively. Baseline demographics included age, BMI, prior surgical and obstetrical history, and menopausal status. Objective measures included pelvic organ prolapse quantification (POPQ) examination and a CST with prolapse reduction using Proctoswabs at the time of baseline multi-channel urodynamic testing. The CST challenge was performed with 250 ml in the bladder. Additionally, Valsalva and cough leak point pressures (LPP) were collected. For the postoperative voiding trials, all Foleys were removed early in the morning and the patient’s voids were measured and reported to the surgeon who would decide to send the patient home with or without a catheter. The decision is based on the voided volumes (a minimum of 200 ml), the time interval since Foley removal or last void, occasional use of a bladder scanner, and the patient’s report of feeling that her bladder was empty. Subjective measures included the urinary subscales of validated condition-specific questionnaires: the Urinary Distress Inventory Short Form (UDI-6) and the Urinary Impact Questionnaire Short Form (UIQ-7).

The primary outcome measure was cure, defined objectively and subjectively at 1 year. Objective cure was defined by a negative standing CST at a volume of 250 ml in the bladder. Subjective cure was defined by an answer of 0 (no) or 1 (yes, not bothersome) on question 17 of the Pelvic Floor Distress Inventory-short form (PFDI-20), which reads, “Do you experience urine leakage related to coughing/sneezing/laughing?” Overall cure included the satisfaction of both objective and subjective criteria; in other words, if a patient did not leak on the CST, but had a positive answer to question 17 of the PFDI-20, then she would be considered a failure and vice versa.

Secondary outcome measures included the change in symptoms and quality of life questionnaires—the Urinary Distress Inventory (UDI-6) and the Urinary Impact Questionnaire (UIQ-7) Short Forms—at 1 year. All patients were evaluated for complications such as cystotomy, mesh erosion, postoperative pain, need for reoperation, and urinary retention.

Statistical analysis included descriptive statistics, and paired Student’s* t* tests for the questionnaire scores; leak point pressure and postvoid residual (PVR) values. The Wilcoxon signed-rank test was used for the pelvic organ prolapse quantification (POP-Q) measures and stages. Statistical significance was defined by an alpha value of 0.05.

## Results

**Fig. 1:**
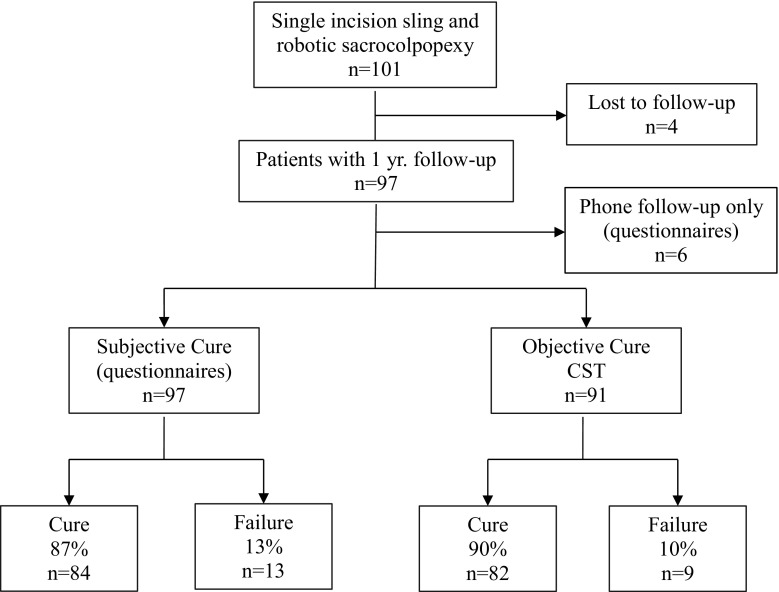
Flowchart of patients

Between August 2010 and July 2012, 101 patients underwent the single-incision sling placement at the time of robotic sacrocolpopexy and were included (Fig. 1). The average age was 56.5 with an average BMI of 26.5 and a median stage III prolapse, as listed in Table 1.

**Table 1:** Baseline demographic data (*N* = 101)

Characteristic	Data
Age (years) mean ± SD	56.5 ± 9.8
BMI (mean ± SD)	26.5 ± 5.1
Vaginal parity (median)	2
Postmenopausal (%)	61.4
Smoker (%)	8
Subjective preoperative urgency (%)	59

One-year follow-up was obtained for 97 out of 101 patients (96 %). Six patients out of the 97 were only available for follow-up over the phone. All patients had a positive CST during their preoperative urodynamic testing. The vast majority (90 out of 101, 89 %) complained of some degree of SUI at intake. Occult SUI upon reduction of the prolapse was discovered in only 11 patients (10.8 %) during their preoperative urodynamic testing. Of 101 patients, 100 (99 %) passed their voiding trial on the first postoperative day and none presented later with urinary retention. One patient required a second day for a successful voiding trial in the office. There were no intraoperative cystotomies, no mesh erosions, no sling revisions, and no cases of urinary retention as listed in Table 2. One postmenopausal patient complained of new onset dyspareunia at the 3-month visit, but this problem resolved with vaginal estrogen.

**Table 2:** Complications (*n* = 91, objective follow-up; *n* = 97, subjective follow-up)

Complication	Data, % (*n*)
New onset dyspareunia	1 (1/97)
Intraoperative cystotomy	0 (0/101)
Mesh erosion	0 (0/91)
Urinary retention	0 (0/97)
Sling revision	0 (0/97)
Pain at 1 year	0 (0/97)

**Fig. 2:**
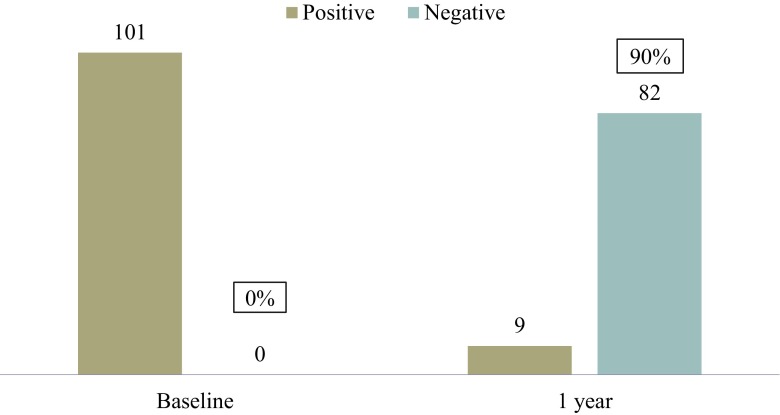
Objective cure based on the cough stress test (CST). All 101 patients at baseline had a positive CST, while 90 % (*n* = 82) were cured at 1 year

Overall cure based on both objective and subjective criteria was 87 % at 1 year. Objective cure based on a negative CST was 90 % (82 out of 91). Subjective cure based on the postoperative PFDI-20 questionnaire was 87 % (85 out of 97; Fig. 2). If the 4 patients who were lost to follow-up, were considered failures then the success rate would be 84 % (85 out of 101). Out of the 12 patients who were not cured, 8 underwent subsequent anti-incontinence procedures (6 retropubic slings and 2 periurethral bulking agents); the remaining 4 did not seek any treatment.

**Fig. 3:**
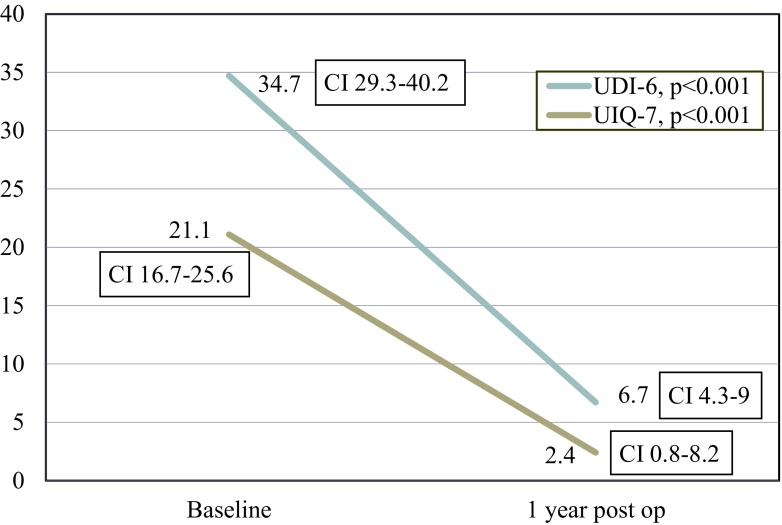
Change in validated quality of life questionnaires, including the Urinary Distress Inventory-short form (*UDI-6*) and the Urinary Impact Questionnaire-short form (*UIQ-7*) from baseline to 1 year postoperatively

The UDI-6 scores improved from 34.8 ± 25.1 at baseline to 6.7 ± 11.2 at 1 year (*p* < 0.001). Similarly, UIQ-7 scores improved from 21.1 ± 22.8 at baseline to 2.4 ± 8.2 at 1 year (*p* < 0.001; Fig. 3). Prolapse cure rate at 1 year was 93.4 % based on the NIH criteria of POP-Q stage 0 or 1. Table 3 includes the POP-Q examination points and prolapse stage before and 12 months after surgery.

**Table 3:** Pelvic organ prolapse quantification (POP-Q) measurements and stage, at baseline and 1 year (*n* = 91)

Prolapse measure	Preoperatively	12 months postoperatively	*P* value*
Aa Median (Q1, Q3)	+1 (0, +2)	−3 (−3, −2.5)	<0.05
Ba Median (Q1, Q3)	+1 (0, +2)	−3 (−3, −2.5)	<0.05
C Median (Q1, Q3)	−1 (−2, +1)	−8 (−9, −8)	<0.05
Ap Median (Q1, Q3)	0 (−1, +1)	−3 (−3, −2)	<0.05
Bp Median (Q1, Q3)	0 (−0.5, +1)	−3 (−3, −2)	<0.05
TVL Median (Q1, Q3)	9 (9, 10)	9.5 (9, 10)	0.936
POP-Q stage Median (Q1, Q3)	3 (2, 3)	0 (0, 1)	<0.05

*Q1* first quartile, *Q3* third quartile, *TVL* total vaginal length

*Wilcoxon signed-rank test

The preoperative baseline LPP on urodynamic testing was lower in patients who continued to leak (12 out of 97) than those who were cured (85 out of 97); 74.2 cm H_2_O (CI 54.4–93.9) vs 102.7 cm H_2_O (CI 94.1–111.3); *p* = 0.01.

There was no difference in PVR at baseline and at 1 year (36.1 ml vs 20.8 ml) respectively (*p* = 0.02).

## Discussion

This study showed that the addition of the Miniarc Precise® single-incision suburethral sling at the time of robotic sacrocolpopexy resulted in a high success rate (87 % at 1 year), without mesh erosions, postoperative vaginal or thigh pain, hematomas, voiding dysfunction or urinary retention.

The strengths of this study include its prospective nature, a high follow-up rate (96 %), and the fact that the patients served as their own controls before and after treatment. The limitation of this study is the lack of a comparison group. The surgeries were performed in a high volume center by a fellowship-trained urogynecologist. Only patients who demonstrated stress incontinence prior to surgery received the treatment.

The fact that 99 % of patients passed their voiding trial on postoperative day 1 highlights the non-obstructive nature of this single-incision sling, despite the fact that it is not a “tension-free” sling. All 6 of the patients who subsequently underwent a retropubic sling placement because of persistent postoperative leakage were “dry” at their latest follow-up. The single-incision sling mesh was not revised at the time of retropubic sling placement and it did not hinder the procedure or its success rate.

Caution should be taken when applying these results to patients without prolapse, as they were not included in this study. The relationship between prolapse and urinary incontinence and their concomitant treatment has been the subject of many studies. The largest randomized trials (the CARE and OPUS trials) to address this issue recommended the routine performance of anti-incontinence procedures at the time of prolapse repair [3, 4]. Despite this evidence, the debate goes on and many studies and publications since have called for a more tailored approach and even offered an online risk calculator for de novo SUI [13]. In the current study occult SUI was discovered in 11 patients (10.8 %) on preoperative CST with prolapse reduction. In The OPUS trial the risk of new onset, postoperative SUI at 3 months was 71.9 % for patients with occult SUI if they did not undergo a sling [4]. At our center we continue to offer a suburethral sling to patients with occult SUI and discuss with them the risks and benefits of a “prophylactic sling” vs a “two-stage” approach. As to the choice of sling, the current study has encouraged some of us to continue to implant the MiniArc® sling at the time of sacrocolpopexy, especially for patients with significant preoperative voiding difficulties. We prefer to use a retropubic sling for patients with intrinsic sphincter deficiency (ISD) or an LPP ≤ 60 cm H_2_O.

The 1-year prolapse cure rate of 93.5 % is consistent with prior publications on robotic sacrocolpopexy [14–16]. This high success rate is important for the validity of the mini-sling results, since a recurrent anterior wall prolapse would have increased the “apparent success rate” of the anti-incontinence procedure in the same way that preoperative prolapse masks occult SUI.

In conclusion, the addition of a single-incision suburethral sling at the time of robotic sacrocolpopexy in women with SUI resulted in a cure rate of 87 % at 1 year. Future long-term and comparative studies are needed to further elucidate the role of the single-incision sling in the treatment of SUI in patients with and without pelvic organ prolapse.
